# Older Male Physicians Have Lower Risk of Trochanteric but Not Cervical Hip Fractures

**DOI:** 10.3390/ijerph120202249

**Published:** 2015-02-16

**Authors:** Hsiu-Nien Shen, Wei-Ting Lin, Chin-Li Lu, Chung-Yi Li

**Affiliations:** 1 Department of Intensive Care Medicine, Chi Mei Medical Center, No. 901 Chung-Hwa Road, Yong-Kang district, Tainan 71004, Taiwan; E-Mail: hsiunian@gmail.com; 2 Department of Public Health, College of Medicine, National Cheng Kung University, Tainan 70101, Taiwan; E-Mail: chinli.lu@gmail.com; 3 Department of Orthopedics, Chi Mei Medical Center, No. 901 Chung-Hwa Road, Yong-Kang District, Tainan 71004, Taiwan; E-Mail: aapriliaa@gmail.com; 4 Department of Medical Research, Ditmanson Medical Foundation Chia-Yi Christian Hospital, No. 539 Zhongxiao East Rd., East district, Chiayi 60002, Taiwan; 5 Department of Public Health, China Medical University, Taichung 40447, Taiwan

**Keywords:** epidemiology, physician, exercise, fracture, cohort studies

## Abstract

*Background*: Osteoporosis is pathophysiologically related to trochanteric fractures, and this condition is more preventable by lifestyle modifications than cervical fractures. We investigated whether older physicians, who are health-conscious people, are at a lower risk of hip fractures because of fewer trochanteric fractures. *Methods*: Data regarding older (≥65 years) physicians (*n* *=* 4303) and matched non-medical persons (control) were retrieved from Taiwan’s National Health Insurance claims. All of the subjects were obtained from NHIRD with index dates from 1 January 2000 to 31 December 2008. Cox proportional hazard and competing risk regression models were established to estimate the hazard ratio (HR) of hip fracture associated with older physicians. *Results*: The incidence rates of trochanteric fractures were lower in older physicians than in controls (1.73 and 3.07 per 1000 person-years, respectively), whereas the rates of cervical fractures were similar between the two groups (2.45 and 2.12 per 1000 person-years, respectively). Older physicians yielded 46% lower hazard of trochanteric fractures than controls (adjusted HR 0.54, 95% confidence interval 0.37–0.79); by contrast, hazards of cervical fractures were comparable between the two groups. The HRs estimated from the competing risk models remained unchanged. *Conclusions*: Our findings indicated that health risk awareness may pose a significant preventive effect on trochanteric hip fractures.

## 1. Introduction

Hip fracture is a major and potentially preventable public health problem. This condition may be prevented by reducing several risk factors of osteoporosis, such as physical inactivity, nutritional deficiencies, and unhealthy lifestyles [1,2,3]. These controllable risk factors are generally known to physicians, who constitute a particularly health-conscious group thus more likely to adopt risk-reducing modifications for the prevention of osteoporosis [4,5,6].

Physicians possibly experience a lower risk of hip fractures than non-medical persons; furthermore, this risk difference is greater in trochanteric fractures than in cervical fractures. This difference is possible because the trochanteric hip site is composed of more cancellous bone with a higher bone turnover than the cervical hip site; thus, the former is more likely affected by lifestyle habits, such as physical activities, than the latter [7,8,9,10,11,12]. However, these hypotheses should be further verified because physicians may not always be able to take the advantage of being a medical professional [13,14,15]. For instance, physicians with diabetes are not at a lower risk of hospitalization for coronary heart disease than those without diabetes [15], suggesting that some barriers may exist and impede physicians’ intention to utilize preventive measures or to manage their health effectively. Moreover, physicians experience a high prevalence of job stress and burnout [13], which may theoretically lead to osteopenia and osteoporosis [14,16].

In addition to concerns on potential occupational safety and health risks of physicians, information on type-specific fracture risks may exhibit important public health implications. The level of hip fracture risk of physicians may be set as a realistic goal for public health policies because these physicians may serve as role models of healthy behaviors for the public [17], if these physicians are indeed at a less risk. Therefore, we conducted this study to compare risks of hip fractures, including cervical and trochanteric types, between older physicians and covariate-matched non-medical individuals.

## 2. Methods

### 2.1. Database

The National Health Insurance of Taiwan is compulsory and covers all citizens except prison inmates. Study subjects were obtained from the National Health Insurance Research Database (NHIRD) [15] and released for research purposes by the National Health Research Institute, Taipei, Taiwan. The NHIRD covers nearly all (99%) inpatient and outpatient claims for Taiwan’s total population of >22 million; furthermore, NHIRD is one of the largest and most comprehensive databases in the world. This database has been used extensively in various studies, including hip fracture [18,19,20,21,22,23] and physicians’ health [15,24,25,26]. The NHIRD provides encrypted patient identification number, gender, birthday, dates of admission and discharge, medical institutions providing health services, *International Classification of Diseases*, *Ninth Revision*, *Clinical Modification* (ICD-9-CM) diagnosis (up to five) and procedure (up to five) codes, outcome at hospital discharge (recovered, died, or transferred), and fees charged to patients. Information on medical personnel (including physicians and other health care workers), including licensed date, specialization, employment area, employment type, and encrypted identification number, which can be linked to claims data, is also available.

### 2.2. Ethics Statement

The review board of the Medical Research Committee in Chi Mei Medical Center approved the study (CMFHR10181) and waived the need for formal ethical approval and written informed consent from the participants because a reimbursement database containing encrypted and transformed data was used.

### 2.3. Study Cohort

The period of this cohort study ranged from 1 January 2000 (index date) to 31 December 2008. We initially identified 4330 physicians aged ≥65 years and received certification before 2000. Non-physician controls, excluding other healthcare workers, were selected from a one-million random sample of beneficiaries in 2000. The physicians who suffered from hip fracture between 1997 and 1999 were excluded (*n =* 27); the remaining physicians were individually matched (1:1) to non-physician controls based on gender, year of birth, urbanization (including urban, suburban, and rural area), and income level (including high, middle, low, and no income defined by insurance premium) [27]. These matching variables were considered to select demographically and socio-economically matched controls. Controls were excluded before matching was performed if the controls suffered from hip fracture between 1997 and 1999. A total of 4303 matched pairs were included in the analysis.

### 2.4. Outcomes

We identified the first episode of non-traumatic hip fracture (ICD-9-CM codes 820) within the study period as primary outcome. Hip fractures were also classified according to site as cervical fractures (ICD-9-CM codes 820–820.19, 820.8, and 820.9) or trochanteric fractures (ICD-9-CM 820.2–820.32) [28]. Hip fractures associated with transport accident (E800–E848) were excluded from the outcome of interest. All of the study subjects were followed from the index date to the occurrence of end point, withdrawal from the insurance (usually due to deaths), or 31 December 2008, whichever date came first. Subjects with the last two conditions were considered censored in the analysis.

### 2.5. Confounders

Considering that medical comorbidity is associated with increased risk of osteoporotic fractures [29,30], we used Charlson’s comorbidity index score (continuous variable) as the main confounder [31,32]. The score is a weighted summary measure of 19 clinically important medical conditions (including neurologic, cardiovascular, pulmonary, renal, hepatic, and autoimmune diseases, as well as dementia, diabetes, cancers, peptic ulcer, and acquired immunodeficiency syndrome); this score is applied in ICD-9-CM coded administrative databases. A high score indicates a great comorbid burden. To calculate the score, we retrieved information on a study subject’s underlying illnesses from inpatient and outpatient claims in 1997–1999.

### 2.6. Statistics

Continuous variables were described as mean (standard deviation), whereas discrete variables were described as counts or percentages. Overall and type-specific incidence rates (IR) and 95% confidence intervals (CI) of hip fractures were determined in accordance with Poisson’s assumption. Differences in responses between physicians and controls were compared by Mann-Whitney’s *U* or Student’s *t* test (for continuous variables, as appropriate) and *χ*^2^ test (for discrete variables). Kaplan-Meier failure estimates of hip fractures were plotted and differences between the two groups were examined by log-rank test. Cox proportional hazard regression model with paired data was used to assess the hazard ratios (HRs) of hip fractures, in which data were adjusted in terms of Charlson’s score. Proportional-hazard assumption was verified using the plots of *log*[–*log*(survival function)] *vs.* *log*(time) and Schoenfeld residuals *vs.* time. Model performance was assessed using Harrell’s *c* statistic. We also evaluated the interactive effect of group and age on hip fracture risks in the regression models.

A relatively high competing mortality risk may overestimate the probability of hip fracture if this competing risk is not adjusted because we have been evaluating older people for a long period [33,34]. Therefore, we further analyzed these individuals by considering withdrawal as a competing risk event to assess the risks of hip fractures. Trochanteric fractures were also considered as competing risk events and vice versa when we modeled cervical fractures. Cumulative incidence functions and sub-hazard ratios (SHR) of competing risk regression were plotted and computed using “*stcurve*” and “*stcrreg*” commands, respectively, in STATA 12.0, which is based on Fine and Gray’s proportional sub-hazard model [35]. Data were analyzed using professional statistical packages, particularly SPSS version 17.0 for Windows (SPSS Inc., Chicogo, IL, USA) and STATA version 12.0 for Windows (StataCorp LP, College Station, TX, USA; www.stata.com). A two-tailed *p* < 0.05 was considered significant.

## 3. Results

### 3.1. Baseline Characteristics

Table 1 shows the baseline characteristics of the study cohort. The mean age of the physicians was 74.1 years (range 65–99 years); most of these physicians were males (97.9%), with low or no income (70.1%), and residing in urban areas (70.5%). The prevalence of comorbidity was significantly lower in physicians than in controls (mean Charlson’s score: 0.56 *vs.* 0.63, *p* < 0.01).

### 3.2. Incidence Rates and Risks of Hip Fractures

Table 2 shows the overall incidence rates, the type-specific incidence rates, and the relative risk estimates of hip fractures. The overall incidence rates of hip fractures of physicians and controls were 4.18 and 5.19 per 1000 person-years, respectively. By contrast, the incidence rates of trochanteric fractures of physicians and controls were 1.73 and 3.07 per 1000 person-years, respectively. Furthermore, the incidence rates of cervical fractures were similar between the two groups (2.45 and 2.12 per 1000 person-years, respectively). Physicians were similar in age when they suffered from hip fractures compared with controls [trochanteric fractures 83.3 *vs.* 82.7 years (*p* = 0.532) and cervical fractures 82.7 *vs.* 83.5 years (*p* = 0.365)].

**Table 1:** Baseline characteristics of older physicians and controls.

Variables	Physicians (*n* = 4303)	Controls (*n* = 4303)	*p* Values
Mean age ± SD	74.1 ± 5.6	74.1 ± 5.6	matched
Male, %	97.9	97.9	matched
Income level, %	--	--	matched
Zero	13.6	13.6	
Low	56.5	56.5	
Middle	5.2	5.2	
High	24.8	24.8	
Urbanization, %	--	--	matched
Urban	70.5	70.5	
Suburban	22.7	22.7	
Rural	6.8	6.8	
Charlson’s score (mean ± SD)	0.56 ± 0.97	0.63 ± 1.02	0.002
0	65.8	62.8	
1	20.2	20.7	
2	9.0	10.1	
≥3	5.1	6.3	

Abbreviations: SD, standard deviation.

**Table 2:** Incidence rates (IR, per 1000 person-years) and hazard ratios of hip fracture in older physicians and controls (*n =* 4303 in each group), 2000–2008.

**Hip Fracture**	**Physicians**		**Controls**	
	***N***	**IR (95% CI)**	***N***	**IR (95% CI)**
All	140	4.18 (3.54–4.93)	169	5.19 (4.44–6.01)
Cervical	82	2.45 (1.96–3.03)	69	2.12 (1.66–2.66)
Trochanteric	58	1.73 (1.33–2.23)	100	3.07 (2.51–3.71)
**Hip Fracture**	**Cox Model**		**Competing Risk Model**	
	**Crude HR (95% CI)**	**Adjusted HR (95% CI) ^a^**	**Crude SHR (95% CI)**	**Adjusted SHR (95% CI) ^a^**
All	0.77 (0.59–0.99)	0.78 (0.60–1.02)	0.83 (0.66–1.03)	0.83 (0.67–1.04)
Cervical	1.07 (0.74–1.55)	1.14 (0.78–1.67)	1.19 (0.86–1.64)	1.20 (0.88–1.66)
Trochanteric	0.55 (0.38–0.79)	0.54 (0.37–0.79)	0.58 (0.42–0.80)	0.58 (0.42–0.82)

^a^ Charlson’s comorbidity score was enrolled as the main confounder. Abbreviations: CI, confidence interval; HR, hazard ratio; SHR, sub-hazard ratio.

**Figure 1:**
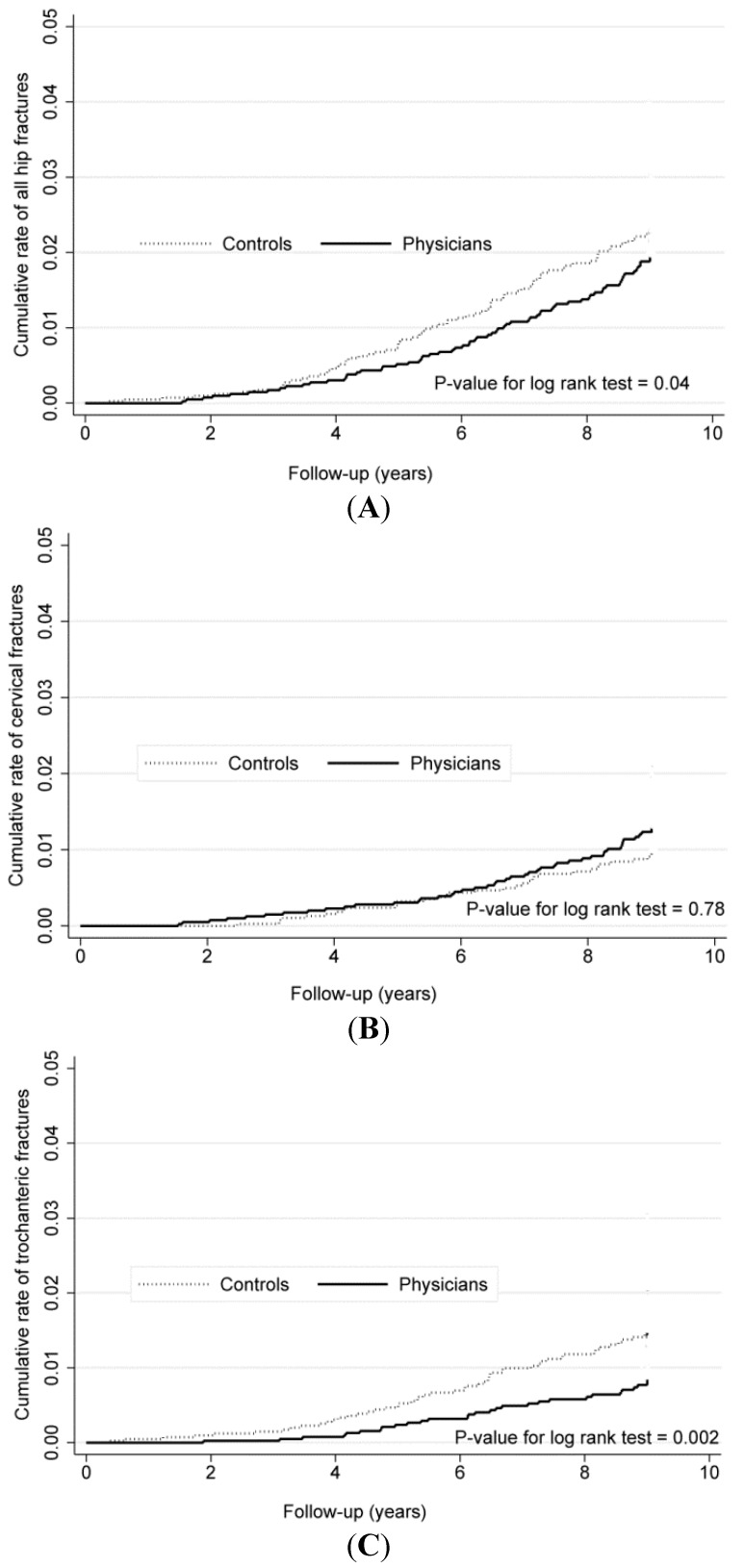
Comparisons of Kaplan-Meier failure estimates of overall (**A**) and type-specific hip fractures (**B**,**C**) between the two groups, 2000–2008.

Figure 1 plots the Kaplan-Meier failure estimates of overall and type-specific hip fractures between the two groups. In the Cox regression models, the crude HR of all hip fractures was 0.77 (95% CI, 0.59–0.99); however, this parameter became statistically non-significant after Charlson’s score was adjusted (Harrell’s *c* = 0.60). In the fracture type-specific analysis, physicians were found to exhibit 46% lower risk of trochanteric fractures (adjusted HR 0.54, 95% CI, 0.37–0.79) but comparable risk of cervical fractures (adjusted HR 1.14, 95% CI, 0.78–1.67). No significant interaction was found between group and age on the risks of hip fractures.

### 3.3. Competing Risk Analysis

**Figure 2:**
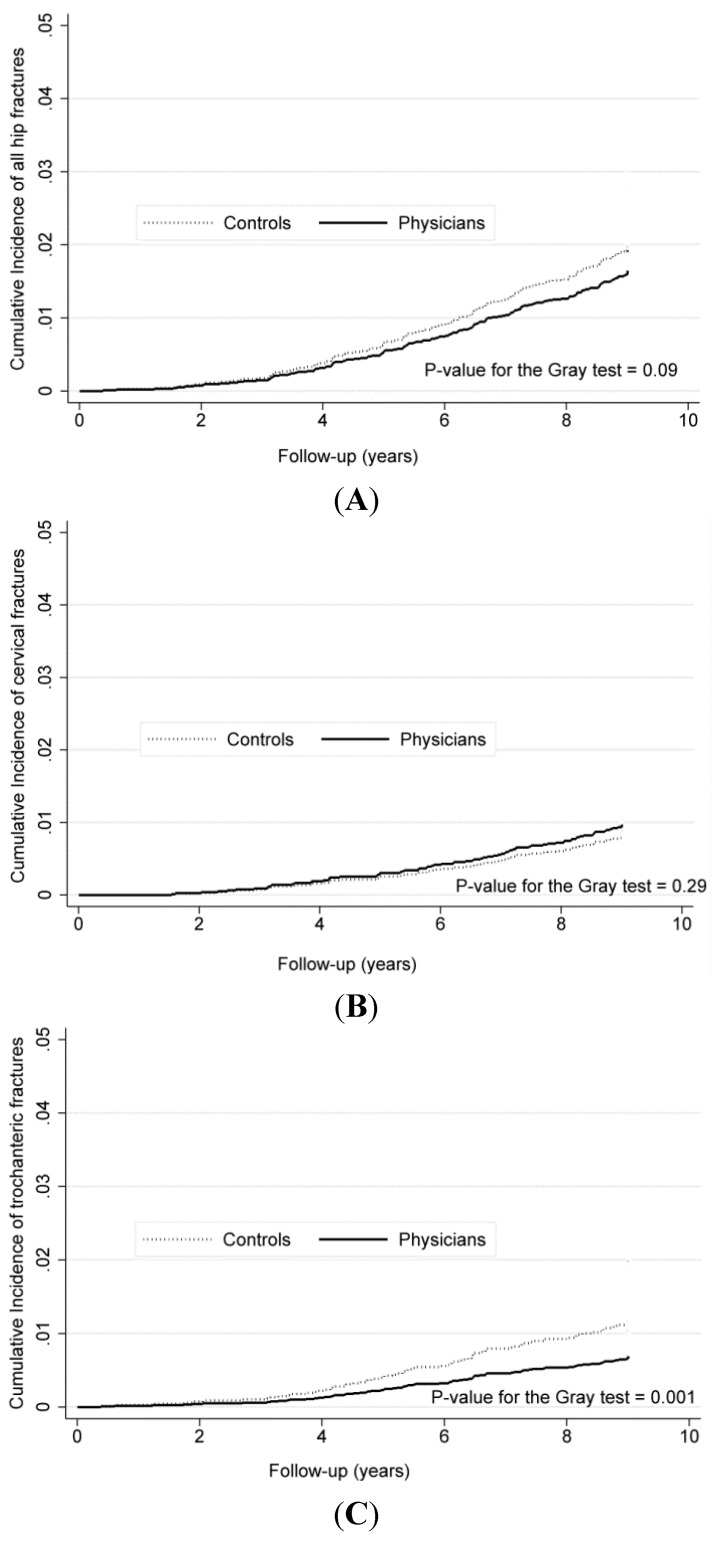
Comparisons of cumulative incidence functions of overall (**A**) and type-specific hip fractures (**B**,**C**) between the two groups, 2000–2008.

Withdrawals occurred in 1246 (29.0%) physicians and 1383 (32.1%) controls. Figure 2 shows the cumulative incidence functions of overall and type-specific hip fractures for the two groups. In the competing risk models, both crude and adjusted SHRs of overall hip fractures were not significant, but the results of type-specific risks were similar between Cox and competing risk models (Table 2).

## 4. Discussion

In this matched cohort study, the risks of overall and type-specific hip fractures in older Taiwanese physicians were assessed. Our results showed that these physicians exhibited a lower risk of trochanteric fractures than controls. Overall and cervical-type fracture risks were similar between the two groups. These findings agree with our hypothesis and imply that awareness of health risks may help prevent the occurrence of trochanteric hip fractures in physicians.

Some of the results of the present study may be compared with a previous report based on Taiwan’s NHIRD [22]. Our study should be more representative of males because approximately 98% of the older Taiwanese physicians were males. The previous study showed that the incidence rates of operated hip fractures in older males with overall, cervical, and trochanteric fractures were 4.84, 2.09, and 2.74 per 1000 person-years, respectively, in 2002 [22]. These figures are slightly lower than the corresponding figures in our control group, particularly 5.19, 2.12, and 3.07 per 1000 person-years, respectively. This difference is possible because all hip fractures were included in the present study, but only operated hip fractures were considered in the previous study [22]. Furthermore, older people were selected from a higher socioeconomic group in our study. However, the effect of socioeconomic status on hip fractures is considered weak because of the small differences in incidence between relatively high-income controls in our study and general population in the previous study [22].Despite these findings, the relation between hip fracture risk and socioeconomic status (in terms of income, education, and occupation) remains uncertain [33]. 

We found that the risk estimates were similar between Cox and competing risk models. Competing mortality risk should be considered in studies on fracture risks in older people when follow-up time is long (>5 years) and competing risk is high (greater than or equal to the proportion of subjects experiencing the outcome of interest). In the presence of informative censoring, failure to account for the competing risk may substantially overestimate the risk of fractures [34,36]. Although the mean follow-up time was 7.7 years and the competing risk occurred in approximately 30% of the subjects in our study, risk estimates in traditional Cox regression models unlikely deviate significantly from those in competing risk models. The consistent results of the two models support our findings.

The results of the study may help policy makers define a realistic goal of preventive measures for hip fractures in older people. This study also revealed different physicians’ risks of cervical and trochanteric fractures; this finding supports our hypothesis stating that separate preventive measures may be required for the two types of hip fractures [9,11]. The trochanteric hip site is composed of more cancellous bone and the cervical hip site is composed of mainly cortical bone; as such, trochanteric fractures are more likely to be prevented by measures that involve an increase in bone mass than cervical fractures [9,11]. Furthermore, physicians show greater awareness of health risks than non-medical practitioners; for this reason, these physicians may serve as role models of hip fracture prevention for the public. Therefore, information on their life habits is likely valuable. However, this information is insufficiently available in Taiwan; as such, we can only refer to reports from other countries [4,5]. For example, surveys in the United States and Canada revealed that physicians exhibit considerably lower prevalence of smoking (3%–6% *vs.* 15%–18%) and drinking (1%–6% *vs.* 9%–23%) than general population [4,5]. Although physicians spend longer work hours than the general population (60% > 40 h per week or average 49 h per week *vs.* average 36.5 h per week), most physicians manage to participate in regular exercise (63% with regular moderate to vigorous exercise of physicians in the US; average 4.7 h of mild to vigorous exercise per week of physicians in Canada) but not as active as general population (74% with regular moderate to vigorous exercise in the US population) [4,5]. The proportions of physicians consuming regular multivitamin or mineral supplements vary between 20% and 35%; these figures are similar to those of women in general population [4,5,6]. These observations indicated that major differences in life habits are observed between physicians and general population in these two countries; hence, life habits can be considerably improved.

Several limitations deserve comments. First, medical profession is only a surrogate measure of knowledge on and awareness of risk factors for osteoporotic fracture. However, level of knowledge is likely varied, which possibly underestimates the observed difference in hip fracture risk. Second, the accuracy of diagnosis codes for hip fractures cannot be validated. Some patients may be miscoded in terms of hip fractures. However, this potential problem is also likely non-differential; hence, observed differences are possibly biased toward the null. Third, some co-morbidities among physicians may have been overlooked because of self-medication, which likely underestimates the observed differences [30]. Fourth, some variables such as diet, body weight and height, life-styles (including smoking and drinking), visual impairment, number of falls, medication, calcium intake, and bone mineral density are not available from the database. Body height is associated with both socioeconomic status [37] and types of hip fracture [9]. Failure to adjust for these potential confounders might result in residual confounding that could explain, at least to some extent, the observed difference in risk of fracture, as some of these unmeasured variables were found to pose confounding on the association between medication use (sedative-hyponotics and SSRI) and hip fractures [38,39]. The risk estimate of trochanteric fracture is less likely affected by failure to adjust for height because socioeconomically matched pairs are compared, and height is more closely related to cervical fractures [9]. Nevertheless, interpretation of our findings is limited because of insufficient information regarding other variables closely related to the risk of osteoporotic fractures. Further studies should be conducted to describe the distribution of these variables in the two groups and to understand the roles of these variables on the reduced risk of trochanteric hip fractures in older physicians. Difference in ethnicity could limit generalizability to other populations, although our study is strengthened by a national population-based design.

## 5. Conclusions

In conclusion, the findings from this study imply that health risk awareness may have a significant preventive effect for trochanteric hip fractures. Nevertheless, residual confounding and factors such as knowledge (level and duration), working environment, colleagues’ communication, life style, nutrition and exercise level might be responsible for the observed risk difference. Despite that, public health interventions may be considered by encouraging people to adopt risk-reducing modifications for osteoporosis; these interventions may significantly affect the prevention of trochanteric hip fractures. However, these measures are unlikely to elicit a similar effect on cervical fractures.
